# Long‐term outcome of Miniature Schnauzers with genetically confirmed demyelinating polyneuropathy: 12 cases

**DOI:** 10.1111/jvim.15861

**Published:** 2020-08-01

**Authors:** Alba Farré Mariné, Nicolas Granger, Coralie Bertolani, Joan Mascort Boixeda, G. Diane Shelton, Alejandro Luján Feliu‐Pascual

**Affiliations:** ^1^ Aúna Especialidades Veterinarias Valencia Spain; ^2^ CVS Referrals, Bristol Veterinary Specialists at Highcroft Bristol UK; ^3^ The Royal Veterinary College, University of London Hatfield UK; ^4^ Hospital Veterinari Canis Palma de Mallorca Spain; ^5^ Ars Veterinaria Barcelona Spain; ^6^ Department of Pathology, School of Medicine University of California and Comparative Neuromuscular Laboratory San Diego California USA

**Keywords:** aphonic bark, megaesophagus, *MTRM13/SBF2*, tomacula

## Abstract

**Background:**

A demyelinating polyneuropathy with focally folded myelin sheaths was reported in 3 Miniature Schnauzers in France in 2008 and was predicted to represent a naturally occurring canine homologue of Charcot‐Marie‐Tooth (CMT) disease. A genetic variant of *MTRM13/SBF2* has been identified as causative in affected Miniature Schnauzers with this polyneuropathy.

**Objective:**

To provide data on the long‐term progression in affected Miniature Schnauzers from Spain confirmed with the *MTRM13/SBF2* genetic variant.

**Animals:**

Twelve Miniature Schnauzers presented between March 2013 and June 2019.

**Methods:**

Only dogs presented with consistent clinical signs and homozygous for the *MTRM13/SBF2* genetic variant were included. Clinical signs, age of onset and presentation, time from onset to presentation, treatment, outcome, and time from diagnosis to final follow‐up were retrospectively reviewed.

**Results:**

The hallmark clinical signs at the time of presentation were regurgitation with radiologically confirmed megaesophagus (11/12) and aphonic bark (11/12) with or without obvious neuromuscular weakness despite electrodiagnostic evidence of appendicular demyelinating polyneuropathy. Age of onset and clinical presentation were 3‐18 and 4‐96 months, respectively. Treatment was mostly symptomatic and consisted of head elevation during meals, antacids, prokinetics, bethanechol, sildenafil, mirtazapine, or some combination of these. During the follow‐up period (7‐73 months), clinical signs were unchanged in (11/12) cases with aspiration pneumonia developing occasionally (6/12) and being the cause of death in 1 dog.

**Conclusions and Clinical Importance:**

Demyelinating polyneuropathy of Miniature Schnauzers tends to remain stable over the long term leading to a good prognosis with preventive feeding measures and symptomatic treatment to control aspiration pneumonia.

**Abbreviations**
CMAP: compound muscle action potential
CMT: Charcot‐Marie‐Tooth
CSF: cerebrospinal fluid
MTMR: myotubularin‐related
NCV: nerve conduction velocities
SBF2: set‐binding factor 2
T4: thyroxine

## 1 INTRODUCTION

Inherited peripheral neuropathies in dogs are a heterogeneous group of degenerative diseases affecting motor or sensory or both and autonomic peripheral nerves. More than 50 inherited neuropathies have been reported in the veterinary literature, for which clinical, electrophysiological, histopathological, and, for some, mode of inheritance data are available.^1^ However, the mode of inheritance often can only be speculated upon and the specific genetic variants rarely are identified.^2^, ^3^ For many inherited peripheral neuropathies in human beings, the known mutations have allowed further characterization of this group of diseases.^3^ The available knowledge about hereditary neuropathies in humans may be useful to identify genetic mutations in affected dogs and vice versa.^1^

A presumptive inherited autosomal recessive demyelinating polyneuropathy was reported in 3 young Miniature Schnauzers in France in 2008.^4^ The affected dogs were presented for clinical signs of laryngeal paralysis or megaesophagus. Although generalized weakness was not clinically detected, electrodiagnostic testing identified a marked decrease in motor and sensory nerve conduction velocities (NCV), indicating involvement of clinically unaffected nerves. Megaesophagus was present in all of the affected French dogs, and the electrophysiological findings were suggestive of a predominantly demyelinating disease. Variable thickness of the myelin sheath with areas of segmental demyelination and multifocal areas of focally folded myelin sheaths, also known as tomacula, were found in peripheral nerve biopsy specimens.^4^ This disease was predicted to represent a naturally occurring canine form of Charcot‐Marie‐Tooth (CMT) disease (specifically CMT1, B1, and 4B2). In veterinary medicine, other neuropathies in dogs such as hypertrophic neuropathy in the Tibetan Mastiff, with characteristic onion bulb formation,^5^, ^6^ and hypomyelinating neuropathy in the Golden Retriever^7^ have been thought to share similarities with specific forms of CMT disease. Additionally, a suspected inherited tomaculous neuropathy has been described in cattle^8^ and a neuropathy has been described in chickens associated with acquired riboflavin deficiency.^9^

Different genes and variants have been implicated in the pathogenesis of hereditary forms of demyelinating neuropathies in humans associated with focally folded myelin sheaths.^10^ The protein products of these mutated genes include the myelin components PMP22, P0, periaxin, proteins regulating myelin gene transcription early growth response 2 and intracellular Schwann cell proteins involved in the synthesis, transport and degradation of myelin proteins, including the myotubularin‐related (*MTMR*) proteins.^4^ Although degenerative polyneuropathies in dog breeds were reviewed in 2011 and compared with CMT,^1^ none of them were found to share the same mutation as a specific form of the human disease. The *MTMR13/SBF2* (set‐binding factor 2) gene has now been implicated in degenerative polyneuropathy in both human beings and the dogs of this previous report.^11^

Our objective was to provide new clinical data on demyelinating polyneuropathy associated with a genetic variant of the *SBF* gene family and describe the long‐term clinical progression in a larger group of affected Miniature Schnauzers diagnosed in Spain. This new information will increase awareness of the disease in the veterinary community, while providing new data on disease progression.

## 2 MATERIALS AND METHODS

### 2.1 Case selection

Between March 2013 and April 2014, Miniature Schnauzers with presumed autosomal recessive demyelinating polyneuropathy of Miniature Schnauzers and with compatible clinical, electrodiagnostic, or histopathologic findings or some combination of these were evaluated at 4 Spanish veterinary hospitals. Genome‐wide association screening using DNA from 10 affected dogs (2 original French cases,^4^ 7 Spanish cases, and 1 Belgian case) and 5 nonaffected siblings identified a variant in intron 19 of the *SBF2* gene^11^ in all affected dogs, and heterozygosity for the mutation in 3/5 of the nonaffected siblings. Comprehensive details of the mutation detection process have been described in a separate study^11^ and included a genome‐wide association study (GWAS) and resequencing of candidate genes.

After gene discovery, additional Spanish dogs evaluated between June 2014 and June 2019 with consistent clinical signs were identified to be homozygous for the *SBF2* genetic variant, and subsequently also were included in our report.

We describe the presenting clinical signs, age of onset, age at presentation, time from onset to presentation, treatment, outcome, and time from diagnosis to final outcome. Follow‐up information was collected during routine reevaluations or telephone interviews with the owners. Where available, blood results, cerebrospinal fluid (CSF) analysis, electrodiagnostic testing, and histopathologic findings also were included.

### 2.2 Electrodiagnostic studies

A Keypoint portable electrodiagnostic unit (Alpine Biomed, Denmark) was used for electromyography (EMG) and motor NCV testing. Electromyography and motor NCV testing were performed under general anesthesia using IM concentric (coaxial) needles as recording electrodes for the EMG and monopolar needles for the motor NCV. Appendicular, axial, cranial, and laryngeal muscles were evaluated by EMG. Motor NCV were recorded for the tibial and ulnar nerves. The F waves and amplitude of compound muscle action potentials (CMAP) after repetitive stimulation also were determined. Reference values were 46 ± 7 m/s for conduction velocities of tibial and ulnar nerves^12^ and 12.8 ± 3.9 to 16.2 ± 4.3 mV and 13.6 ± 4 to 20.2 ± 5.3 mV for the CMAP amplitudes of the ulnar and tibial nerves, respectively.^13^ A nonaffected sibling was used as a matched control.

### 2.3 Histopathology and histochemistry of muscle and peripheral nerve biopsy specimens

Muscle and nerve biopsy specimens from the cranial tibial muscle and common peroneal nerve, respectively, were collected under general anesthesia after electrodiagnostic testing. Immediately after collection, unfixed chilled muscle and formalin‐fixed muscle were shipped by a courier service to the Comparative Neuromuscular Laboratory, University of California, San Diego. Upon receipt, unfixed muscle was snap frozen in isopentane precooled in liquid nitrogen and stored at −80°C until further processed. Formalin‐fixed muscle was processed by standard methods into paraffin. Light microscopic evaluation of histological and histochemical stains and reactions was performed according to standard protocols^14^ and included hematoxylin and eosin, modified Gomori trichrome, periodic acid Schiff, phosphorylase, esterase, myofibrillar ATPase reactions with preincubation pH of 9.8 and 4.3, reduced nicotinamide adenine dinucleotide‐tetrazolium reductase, succinic dehydrogenase, acid and alkaline phosphatase, and oil red O.

Specimens from the peroneal nerve were immersion fixed in 10% buffered formalin before shipment to the Comparative Neuromuscular Laboratory. Upon receipt, nerves were postfixed in 1% aqueous osmium tetroxide for 3 hours and then dehydrated in a graded alcohol series and propylene oxide. After infiltration with a 1:1 mixture of propylene oxide and araldite resin for 4 hours, nerves were placed in 100% araldite resin overnight and then embedded in fresh araldite resin. Thick sections (1 μm) were cut and stained with paraphenylediamine before light microscopic evaluation.

## 3 RESULTS

### 3.1 Clinical presentation, clinical pathology results, and additional testing

Twelve Miniature Schnauzers (5 intact males, 4 intact females, 1 neutered male, and 2 neutered females) were included. Median age of onset was 12 months (range, 3‐18 months) and median age at presentation was 25 months (range, 4‐96 months). Median time from onset of clinical signs to presentation was 11 months (range, 0‐84 months). Regurgitation associated with megaesophagus and aphonic bark were the most frequent clinical signs in 11/12 cases. Other inconsistently present clinical signs were slightly delayed conscious postural reactions (6/12) and weak flexor reflex in the pelvic limbs (3/12). One dog had muscle tremors in the pelvic limbs and decreased palpebral reflex, and another dog had exercise intolerance. One of the included dogs also presented with seizures, decreased left menace response and delayed left‐sided postural reactions suggesting an independent right forebrain lesion. Computed tomography of the brain with IV contrast medium and cisternal CSF analysis were performed and found to be normal. Thus, epilepsy of unknown origin was suspected. These findings are summarized in Supplemental Information.

Blood evaluations including CBC and serum biochemistry profile (8/12), serum thyroxine and thyroid stimulating hormone concentration (3/12), adrenocorticotropic hormone stimulation test (3/12), and dynamic bile acid stimulation test (1/12) were performed and results were within reference ranges, except for 1 dog that had an increased serum cholesterol concentration. Acetylcholine receptor antibody titers were determined in 2/12 dogs and were within the reference range. Echocardiography and a Holter evaluation in 2 of the dogs were normal. In 2 additional dogs, lumbar CSF analysis was within the reference range.

### 3.2 Electrodiagnostic studies

Electrodiagnostic studies were performed in 5/12 dogs on the right tibial (2/5), left tibial (4/5), right ulnar (1/5), and left ulnar (2/5) nerves. The median motor NCV was 29.55 m/s (range, 23.2‐35.9 m/s) in the right tibial nerve and 26.8 m/s (range, 20‐52.2 m/s) in the left tibial nerve. The motor NCV in the sole right ulnar nerve tested was 24.4 m/s proximally and the motor NCV in the left ulnar nerves tested were 36.7 m/s (1/2) and 18.2 m/s (1/2), both proximally. The CMAP amplitude was measured in 2 right tibial nerves and found to be 4.4 mV proximally and 4.6 mV distally, and 6.1 mV proximally and 3.4 mV distally, respectively; in 3 left tibial nerves it was found to be 5.5 mV proximally and 6.4 mV distally, 4.2 mV proximally and 11.7 mV distally, and 11 mV proximally and 16.6 mV distally; and in 1 right ulnar it was found to be 9.0 mV proximally and 12.4 mV distally. The shape of the curve was polyphasic in 3/5. In the nonaffected sibling used as an age‐matched control, the right tibial and left ulnar nerves were evaluated. Motor NCV was 55.9 m/s proximally and 71.4 m/s distally for the right tibial nerve, and 55.7 m/s for the left ulnar nerve. The CMAP amplitude was 18.2 mV proximally and 24.1 mV distally for the right tibial nerve and 17.7 mV proximally and 17.1 mV distally for the left ulnar nerve.

**FIGURE 1:**
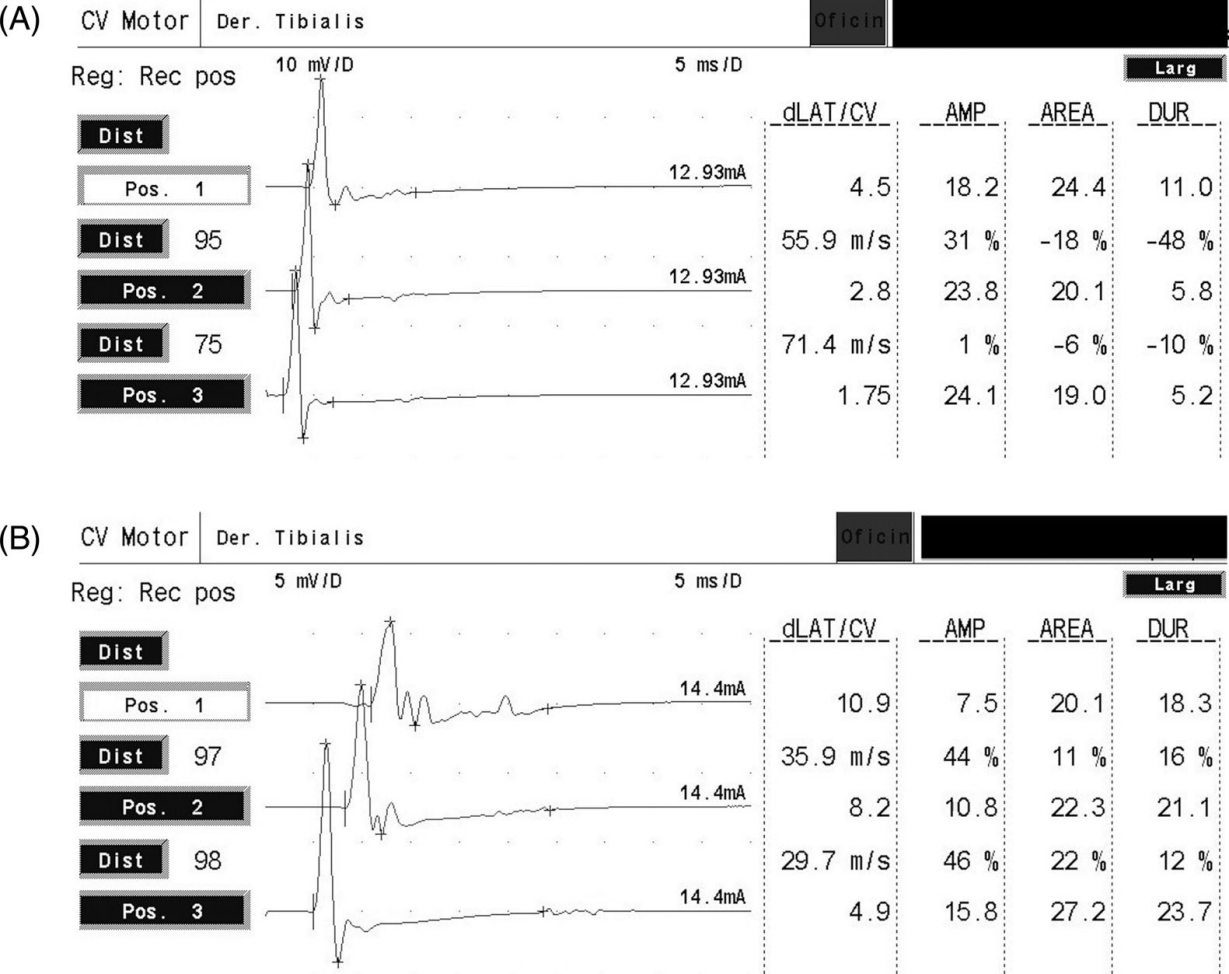
Motor nerve conduction velocities (NCV) of the right tibial nerve of a nonaffected dog (A) and affected dog (B) of the same litter. Note the slow motor NCV (range, 20‐42 m/s) and polyphasic and low compound muscle action potential (CMAP) amplitudes in the affected dog in contrast with the nonaffected dog

In summary, the most prominent abnormalities were slow motor NCV (range, 20‐42 m/s) and low CMAP amplitudes (range, 4.2‐46.0 mV) associated with polyphasic CMAPs (Supplemental Information). These electrophysiological findings were suggestive of a demyelinating disease of the peripheral nerves tested (Figure 1).

### 3.3 Histopathology and histochemistry

**FIGURE 2:**
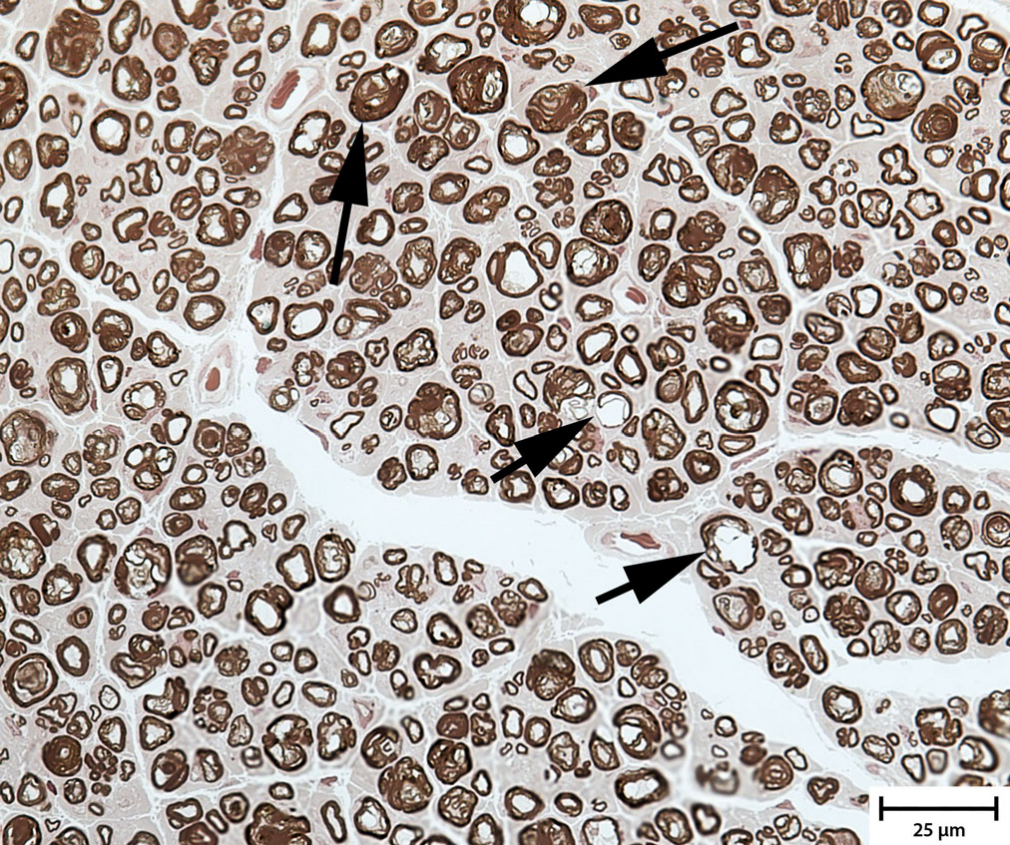
Biopsy specimen from the peroneal nerve (1‐μm‐thick resin sections). Characteristic changes include nonconcentric myelin protrusions around nerve fibers consistent with tomacula (arrows with short tails) and inappropriately thin myelinated fibers (arrows with long tails). Axonal degeneration was not a feature

Muscle and peripheral nerve biopsy specimens were obtained in 1 affected Miniature Schnauzer dog. No specific abnormalities were identified in the biopsy specimens from the cranial tibial muscle. The density of myelinated nerve fibers was subjectively appropriate in the peroneal nerve. Numerous inappropriately thin myelinated fibers and scattered hypermyelinated fibers consistent with tomacula were observed (Figure 2). These findings were consistent with those described in the previous report.^4^

### 3.4 Genetic testing

All of the included cases were tested for the same splicing mutation in intron 19 of the *MTMR13/SBF2* ^11^ gene and found to be homozygous for the mutation. In a litter of 5 2‐month‐old dogs, 2 of the litter were found to be carriers (heterozygous for the mutation) and the other 3 had 2 normal copies of the gene.

### 3.5 Treatment and outcome

Treatment was aimed at ameliorating clinical signs and consisted of head elevation during and 30 minutes after meals (12/12), as well as antacids (omeprazole [3/12] and famotidine [1/12]), intestinal protectants (sucralfate [2/12] and amalgate [1/12]), prokinetics (metoclopramide [3/12], cinitapride [1/12] and mirtazapine [3/12]), and antiemetics (maropitant [2/12]). In 3 cases, sildenafil was used as adjunctive treatment. Bethanechol also was used in 2/12 cases. The frequency of regurgitation remained unchanged in all dogs except in 2 of those receiving mirtazapine, in which episodes decreased from several times daily to once every 2 to 3 days. In the dog with seizures, phenobarbitone was added to the treatment regimen. When aspiration pneumonia developed (6/12), antibiotics also were prescribed (amoxicillin‐clavulanate acid, 4/12; enrofloxacin, 1/12; and cephalexin, 1/12).

Median time from diagnosis to final follow‐up was 22.5 months (range, 7‐73 months). Regardless of treatment, clinical signs slowly progressed in 1/12 dogs leading to clinically evident pelvic limb weakness, muscular atrophy, decreased flexor reflexes, and delayed postural reactions 67 months after diagnosis. In the other dogs, clinical signs were unchanged for 0 to 88 months with aspiration pneumonia developing occasionally in 6 dogs. Four dogs were dead at the time of writing. Death was directly attributed to the disease in only 1 dog after developing aspiration pneumonia 73 months after diagnosis. One dog was euthanatized because of acute renal failure caused by *Leishmania* spp. infection 36 months after diagnosis. Another dog died after a short period of anorexia 17 months after diagnosis, and the cause of death in the last dog 26 months after diagnosis was unknown.

## 4 DISCUSSION

Charcot‐Marie‐Tooth disease constitutes a large group of hereditary motor and sensory peripheral neuropathies in humans characterized by phenotypic and genetic heterogeneity, which can be inherited as autosomal dominant, autosomal recessive or X‐linked traints. Clinical, electrophysiological and pathological features allow differentiation of 3 main subtypes: demyelinating (CMT1), axonal (CMT2), and intermediate (I‐CMT) forms.^15^ To date, genetic variants in >24 genes have been found to cause different forms of CMT disease in people.^10^ Inherited motor and sensory neuropathies in 22 dog breeds have been reviewed and suggested to have a link with CMT subtypes in humans. However, in contrast with CMT, all of the described inherited polyneuropathies in dogs seem to share an autosomal recessive mode of inheritance, although not confirmed by pedigree analysis. It is also difficult to clearly differentiate between demyelinating and axonal neuropathies, and onion bulb formation is infrequent in dogs.^1^

In humans, mutations in the *SBF2* gene causes CMT4B2. The *SBF2* gene encodes for a pseudophosphatase of the MTMR protein family, which has a role in regulating vesicular trafficking in Schwann cells. Loss of this protein may lead to uncontrolled folding of myelin, producing the typical focally folded myelin sheaths or tomacula seen in CMT4B^15^ and in affected Miniature Schnauzer dogs. Despite dogs and humans sharing a mutation in the same gene (*SBF2*) and having a similar histopathologic appearance of the nerves, the clinical phenotype differs substantially between the human and the canine forms. Regurgitation and aphonic bark because of megaesophagus and laryngeal paralysis predominate in dogs, and pelvic limb weakness is rare. On the other hand, appendicular limb weakness and decreased reflexes dominate the human phenotype. In human medicine, it is well known that different genetic variants in the same gene can give rise to markedly different phenotypes.^16^ Indeed, involvement of bulbar and facial nerves resulting in vocal cord paresis or diaphragm weakness most frequently has been reported in the CMT4B1 form (caused by a mutation in the *MTMR2* gene) or other CMT types, but not in CMT4B2.^15^, ^16^, ^17^, ^18^ In 1 report of a patient with CMT disease, esophageal and gastric smooth muscle function impairments were observed, but this clinical scenario is not usually described.^19^ Substantial divergence in clinical signs between affected humans and dogs may be related to the striated muscle of the esophagus in dogs as compared to smooth muscle in humans. Theoretically, CMT disease in dogs may be dominated by striated muscle dysfunction.^19^

The CMT4B2 disease is characterized by autosomal recessive inheritance mode and often affects children between 4 and 13 years of age (mean age, 8 years).^15^ This observation is comparable to the findings in the affected Miniature Schnauzers with a young age of onset (between 3 and 18 months of age). Electrodiagnostic findings are characterized by a marked decrease in NCV and amplitude, and prolonged distal latency. Although not evaluated in the Miniature Schnauzer dogs, sensory NCV also are severely decreased. Fibrillation potentials, positive sharp waves and a decrease in the recruitment pattern of motor unit potentials are found in people during needle electromyography. Polyphasic motor unit potentials and giant action potentials can be present in distal muscles.^15^ In the dogs reported here, slow motor NCV (range, 20‐42 m/s), as well as polyphasic and low CMAP amplitudes were found. In people, increased protein concentration (up to 280 mg/dL) may be found in lumbar CSF, despite normal cell counts.^15^, ^20^ Unfortunately, CSF was only evaluated in 3 dogs and results were within reference ranges.

Histopathological findings in CMT4B2 are characterized by a severe loss of myelinated nerve fibers with the remaining fibers generally hypomyelinated, hypermyelinated or unmyelinated. The pathological hallmark is the presence of nerve fibers with focal regions where myelin is thrown into irregular protrusions from the outer myelin sheath, known as myelin outfoldings or tomacula. Inward protrusions, which extend towards the axon, also are present and referred to as myelin infoldings. The protrusion may contain axons or exclusively Schwann cell cytoplasm. Supernumerary Schwann cell processes or basal lamina structures may encircle some fibers (so‐called onion bulbs) indicating the occurrence of repeated episodes of demyelination and remyelination, and abnormal proliferation.^21^, ^22^, ^23^ Muscle biopsy has been performed in a few patients and has identified only minor abnormalities compatible with neurogenic atrophy.^15^, ^17^ In the sole dog in our series in which muscle and nerve biopsies were performed, and in the French dogs previously reported,^4^ minimal to no changes were identified in muscle biopsy specimens and inappropriately thin myelinated nerve fibers and scattered hypermyelinated fibers (tomacula) were identified.

In human medicine, the diagnosis of CMT4 subtypes is based on clinical findings, neurophysiologic studies, family history, and molecular genetic testing that ultimately permit the diagnosis.^24^ Treatment is symptomatic and includes special shoes or ankle or foot orthoses or both to correct foot drop and aid walking; surgery as needed for severe *pes cavus*; forearm crutches, canes, and wheelchairs as needed for mobility; exercise as tolerated; and treatment of pain, depression, sleep apnea, and restless leg syndrome.^24^ In cases of vocal cord paralysis, treatment may include cordotomy, vocal fold lateralization or medialization to preserve speech, and tracheotomy.^19^ One patient described with CMT syndrome and gastrointestinal involvement was treated with promethazine, metoclopramide, and ondansetron, which helped partially.^25^ In the dogs reported here, treatment was mostly symptomatic and consisted of head elevation during meals, antacids, prokinetics, and bethanechol resulting in little improvement in the frequency of regurgitation. Sildenafil has been shown to decrease regurgitation frequency and increase weight gain in dogs with idiopathic megaesophagus^26^ but was used in only 3 of our cases and resulted in no short‐term improvement, although owners withdrew the medication in 2 dogs less than a month after initiation. On the other hand, bethanechol has been shown to increase the amplitude of esophageal contractions.^27^ The lack of any clinically relevant improvement in our cases and those reported in the literature could be a result of different etiology of the megaesophagus in the dogs treated with bethanechol or sildenafil. Also, metoclopramide and cisapride have been shown to be ineffective and can even aggravate clinical signs.^28^, ^29^ The antidepressant mirtazapine was used in 3 cases, resulting in a slight decrease in the frequency of regurgitation in 2 of them, but the low number of dogs receiving this medication precludes any conclusion on its efficacy. The use of different treatments in our retrospective study, in addition to the low number of cases, represent the major limitations of our study. Additional studies are needed to evaluate the efficacy of individual treatments in miniature Schnauzers with this demyelinating polyneuropathy. Novel treatments such as ascorbic acid,^30^, ^31^, ^32^, ^33^ progesterone antagonists (onapristone^34^ and lonaprisan^35^), curcumin^36^, ^37^ and pharmacological inhibition of histone deacetylase 6^38^ are being studied in human medicine,^39^ but although >80 CMT‐causing genes have been identified to date, an effective treatment has not yet been developed for these diseases in people.^40^

Animal models (primarily in mice and *Drosophila* spp.) representing the most frequent forms of CMT in humans are now available,^40^, ^41^ but none is a spontaneously occurring disease. The disease described here is the first spontaneously and naturally occurring demyelinating inherited CMT polyneuropathy in a large animal model with a confirmed genetic variation. However, the differences in phenotype compared to humans might pose a limitation to the use of affected Schnauzers as an animal model for the human disease. However, identification of a treatment that slows progression of the disease in affected dogs still could be valuable for testing in a clinical trial of affected humans.

In CMT disease, life expectancy is not known to be altered in most cases, with some patients presenting in infancy or early childhood with inability to ambulate and weakness of proximal and distal muscles. In some cases, however, the neuropathy can lead to restrictive pulmonary impairment, vocal cord dysfunction or sleep disturbances,^19^ shortening lifespan, with death occurring in the fourth or fifth decade, mostly as a result of respiratory failure.^31^ In the cases described here, despite lack of an effective treatment, clinical signs remained unchanged in all but 1 dog for up to 73 months, with aspiration pneumonia developing occasionally (6/12). Only1 dog progressed slowly to clinically evident pelvic limb weakness 67 months after diagnosis, but without impairment in quality of life. Although 4 dogs were dead at the time of writing, only 1 death was directly attributed to the disease after development of aspiration pneumonia. This finding demonstrates that the demyelinating polyneuropathy of Miniature Schnauzers tends to remain stable, leading to a fair to good prognosis if aspiration pneumonia and anorexia can be avoided or managed.

## CONFLICT OF INTEREST DECLARATION

Authors declare no conflict of interest.

## OFF‐LABEL ANTIMICROBIAL DECLARATION

Authors declare no off‐label use of antimicrobials.

## INSTITUTIONAL ANIMAL CARE AND USE COMMITTEE (IACUC) OR OTHER APPROVAL DECLARATION

Authors declare no IACUC or other approval was needed.

## HUMAN ETHICS APPROVAL DECLARATION

Authors declare human ethics approval was not needed for this study.

## Supporting information


**AppendixS1**: Supporting Information
