# Supplementary material for: Long‐term outcome of Miniature Schnauzers with genetically confirmed demyelinating polyneuropathy: 12 cases
Source: J Vet Intern Med. 2020 Aug 1;34(5):2005–11. doi: 10.1111/jvim.15861 (PMC7517849; doi:10.1111/jvim.15861)
Supplement: Supplementary file 1 — AppendixS1: Supporting Information [file JVIM-34-2005-s001.pdf]

Supplemental Table 1. Clinical signs and progression

Case	Sex	Age of onset (m)	Age at diagnosis (m)	Neuro exam at diagnosis	Aphonic bark	Megaesophagus	Progression	Follow-up time (m)
1	FI	3	4	Slightly delayed conscious postural reactions PLs (Rt>Lt) and weak flexor reflex in Rt PL	Yes	Yes	Stable Occasional aspiration pneumonia	16
2	MI	15	17	Normal	Yes	Yes	Stable until death due to unknown cause	26
3	MI	12	20	Muscle tremors in PLs, decreased palpebral reflex and slightly delayed conscious	Yes	Yes	Stable Occasional aspiration pneumonia	19

				**postural reactions in Lt PL**				
**4**	**FI**	**12**	**96**	**Seizures, decreased Lt menace response and delayed conscious postural reactions Lt TL and both PLs**	**Yes**	**Yes**	**Stable until euthanasia due to anorexia**	**17.5**
**5**	**FN**	**18**	**36**	**Slightly delayed conscious postural reactions PLs (Rt>Lt) and weak flexor reflex in Rt PL**	**Yes**	**Yes**	**Stable but died due to aspiration pneumonia**	**73.25**
**6**	**MI**	**15**	**15**	**Delayed conscious postural reactions in PLs and decreased**	**Yes**	**Yes**	**Stable Occasional aspiration pneumonia**	

				**flexor reflex in PLs**				**67**
**7**	**MI**	**11**	**15**	**Normal**	**No**	**Yes**	**Stable**	**72**
**8**	**MI**	**12**	**72**	**Exercise intolerance**	**Yes**	**No**	**Stable until euthanasia due to renal failure secondary to Leishmania spp. infection**	**36**
**9**	**FI**	**3**	**30**	**Normal**	**Yes**	**Yes**	**Stable**	**46**
**10**	**FI**	**13**	**14**	**Slightly delayed conscious postural reactions Lt PL**	**Yes**	**Yes**	**Stable Occasional aspiration pneumonia**	**11.5**
**11**	**FN**	**6**	**34**	**Normal**	**Yes**	**Yes**	**Stable**	**7**
**12**	**MN**	**14**	**30**	**Normal**	**Yes**	**Yes**	**Stable Occasional aspiration pneumonia**	**17**

F: female, I: intact, M: male, m: months, N: neutered, PL: pelvic limb, TL: thoracic limbs, Rt: right, Lt: left.

Supplemental Table 2. Electrodiagnostic findings

Case	EMG	CMAP shape	Evaluated nerve		NCV (m/s)	CMAP Amplitude (mV)
1	PSW and FP in laryngeal muscles and slight appendicular	Normal	Rt Tibial	Proximal	35.9	4.4
				Distal	29.7	4.6
			Lt Ulnar		36.7	NE
4	PSW and FP in laryngeal muscles	Polyphasia	Lt Tibial	Proximal	25.0	5.5
				Distal	24.5	6.4
5	PSW and FP in laryngeal muscles	Polyphasia	Lt Tibial	Proximal	28.8	4.2
				Distal	21.4	11.7
			Rt Tibial	Proximal	23.2	6.1
				Distal	29.4	3.4
			Lt Ulnar		18.2	NE
6	Normal	Polyphasia	Lt Tibial	Proximal	42.0	11.0
				Distal	28.6	16.6
			Rt Ulnar	Proximal	22.4	9.0
				Distal	NE	12.4
7	FP in plantar and palmar interosseous muscles	Normal	Lt Tibial	Proximal	52.2	1.1
				Distal	20	NE

**Matched control (case 1 non-affected littermate)**	**Normal**	**Normal**	**Rt Tibial**	**Proximal**	**55.9**	**18.2**
				**Distal**	**71.4**	**24.1**
			**Lt Ulnar**	**Proximal**	**57.7**	**17.3**
				**Distal**	**NE**	**17.1**

CMAP: compound motor action potential, EMG: electromyography, FP: fibrillation potentials, Lt: left, Rt: right, NCV: nerve conduction velocity, NE: not evaluated, PSW: positive sharp waves.
